# Antigenic reactivity of *Leishmania* (*Viannia*) *lainsoni* axenic amastigote proved to be a suitable alternative for optimizing Montenegro skin test

**DOI:** 10.1186/s13071-024-06486-0

**Published:** 2024-09-27

**Authors:** Leonardo Viana de Melo, Thiago Vasconcelos dos Santos, Patrícia Karla Ramos, Luciana Vieira Lima, Marliane Batista Campos, Fernando Tobias Silveira

**Affiliations:** 1 Parasitology Department, Evandro Chagas Institute (Surveillance Secretary of Health and Environment, Ministry of Health), Ananindeua, Pará Brazil; 2 Tropical Medicine Nucleus, Federal University of Pará, Belém, Pará Brazil

**Keywords:** *Leishmania* (*V.*) *lainsoni*, Axenic amastigote antigen, *Leishmania* (*V.*) *braziliensis*, Axenic promastigote antigen, Montenegro skin test, Laboratory diagnosis, American cutaneous leishmaniasis

## Abstract

**Background:**

Laboratory diagnosis of American cutaneous leishmaniasis (ACL) requires a tool amenable to the epidemiological status of ACL in Brazil. Montenegro skin test (MST), an efficient immunological tool used for laboratory diagnosis of ACL, induces delayed-type hypersensitivity (DTH) response to the promastigote antigens of *Leishmania*; however, human immune responses against infection are modulated by the amastigote of the parasite. *Leishmania* (*V*.) *lainsoni* induces strong cellular immunity in humans; therefore, the antigenic reactivity of its axenic amastigote (AMA antigen) to MST was evaluated for the laboratory diagnosis of ACL.

**Methods:**

Among 70 individuals examined, 60 had a laboratory-confirmed diagnosis of ACL; 53 had localized cutaneous leishmaniasis (LCL), and 7 had mucosal leishmaniasis (ML). Patients were treated at the Evandro Chagas Institute’s leishmaniasis clinic, Pará State, Brazil. Ten healthy individuals with no history of ACL (control group) were also examined. *Leishmania* (*V*.) *braziliensis* promastigote antigen (PRO) was used to compare the reactivity with that of AMA antigen. Paired Student’s t-test, kappa agreement, and *Spearman* test were used to evaluate the reactivity of AMA and PRO.

**Results:**

The mean reactivity of AMA in ACL patients was 19.4 mm ± 13.3, which was higher (*P* < 0.001) than that of PRO: 12.1 mm ± 8.1. MST reactivity according to the clinical forms revealed that AMA reactivity in LCL and ML, 18.8 mm ± 13.3 and 24.3 mm ± 13.7, was higher (*P* < 0.001) than that of PRO, 11.8 mm ± 8.2 and 14.6 mm ± 8.4, respectively.

**Conclusion:**

AMA reactivity was higher than that of PRO, indicating that AMA is a promising alternative for optimizing MST in the laboratory diagnosis of ACL.

**Graphical abstract:**

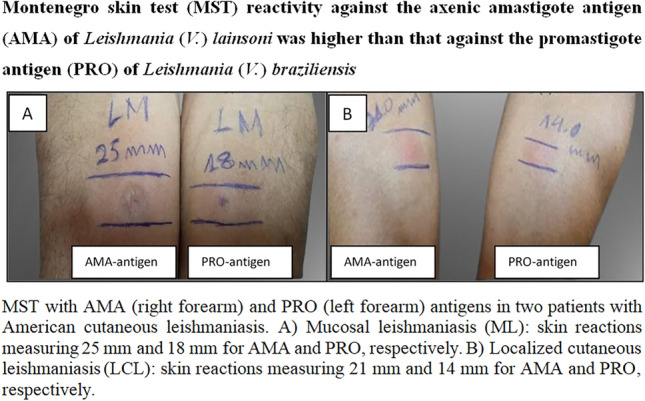

## Background

American cutaneous leishmaniasis (ACL) is an infectious, non-contagious disease caused by at least 15 different species of protozoan parasites of the genus *Leishmania* Ross 1903. *Leishmania* can be taxonomically classified within the following subgenera: *Leishmania* (*Leishmania*) Ross 1903, *L*. (*Viannia*) Lainson & Shaw 1987, and *L*. (*Mundinia*) Shaw, Camargo & Teixeira 2016. From the clinical-immunopathological perspective, ACL can be considered one of most complex parasitic diseases owing to the interaction of different *Leishmania* species with the human immune system [1–3].

ACL is characterized by impairment of the cutaneous and/or mucous tissue. Consequently, ulcerated skin lesions are the major clinical manifestation of the disease. Other types of skin lesions, such as papules, tubercles, nodules, infiltrated plaques, and verrucous or keloid-vegetating lesions, have also been observed. Metastatic mucosal lesions are seen on the nose, mouth, or pharynx alone or simultaneously. Nevertheless, these lesions, with an ulcerogranulomatous appearance, are observed more frequently on the nose and can reach the cartilaginous tissue depending on its depth, leading to nasal septum perforation [4–7].

Seven species of *Leishmania* and a hybrid leishmanial parasite have been recognized as etiological agents of ACL in Brazil, especially in the Brazilian Amazon. This comprises six species of *Leishmania* (*Viannia*) subgenus, including *L.* (*V*.) *braziliensis*, *L.* (*V*.) *guyanensis*, *L.* (*V.*) *shawi*, *L.* (*V.*) *lainsoni*, *L.* (*V.*) *naiffi, L.* (*V*.) *lindenbergi*, a hybrid parasite, *L.* (*V.*) *guyanensis/L.* (*V.*) *shawi*, and only one species of *L*. (*Leishmania*) subgenus, *L.* (*L.*) *amazonensis* [8]. The clinical-immunopathological spectrum of ACL resulting from the interaction of these *Leishmania* species with the human immune system includes four clinical forms: localized cutaneous leishmaniasis (LCL), borderline disseminated cutaneous leishmaniasis (BDCL), mucocutaneous leishmaniasis/mucosal leishmaniasis (MCL/ml), and anergic diffuse cutaneous leishmaniasis (ADCL) [9–11].

Parasitological, histopathological, molecular (mainly the polymerase chain reaction, PCR), and immunological (cellular and/or humoral) methods have been used for the laboratory diagnosis. However, these methods have advantages and disadvantages [12–15]. Parasitological and histopathological methods depend on the parasite load in the skin and/or mucosal lesions. The molecular method requires a complex laboratory infrastructure and highly qualified staff. The immunological method can be used to detect cellular or humoral immune responses. The expression of the cellular immune response is associated with immunological protection in ACL [6, 7]. The immunological method of choice for ACL laboratory diagnosis is based on detecting a specific delayed-type hypersensitivity (DTH) response against *Leishmania* antigens [16–18]. The Montenegro skin test (MST), conceived in the 1920s [19], is a laboratory tool capable of confirming the presence of a DTH response against *Leishmania* infection, which generally indicates a good prognosis [9–11, 20, 21].

The Ralph Lainson Leishmaniases Laboratory (RL/LL) of the Parasitology Department, Evandro Chagas Institute, Secretary of Health and Environment Surveillance, Ministry of Health, Ananindeua, Pará State, Brazil, introduced the use of MST for the laboratory diagnosis of ACL using *L.* (*L.*) *amazonensis* promastigote antigen almost 50 years ago owing to its easy adaptation and laboratory cultivation, which facilitated its standardization [22]. This tool was used until the end of the 1990s in a satisfactory and safe manner.

After new findings provided by the lymphocyte proliferation assay in patients with ACL in the Brazilian Amazon showing that *L.* (*V.*) *braziliensis* promastigote antigen was able to induce greater lymphocyte proliferation rates than those by *L. (L.) amazonensis* promastigote antigen [23], it was decided to replace the *L. (L.) amazonensis* promastigote antigen used in the MST with the *L. (V.) braziliensis* promastigote antigen under the same standardized production and conservation procedures [22]. This change has facilitated a greater range (reaction diameter in millimeters) of MST reactivity since *L.* (*Viannia*) spp. parasites have been recognized as the main agents of ACL in the region, showing greater affinity and specificity for the *L.* (*L*.) *braziliensis* than *L.* (*L*.) *amazonensis* antigen [3, 9, 10].

The development of new laboratory methods capable of promoting axenic amastigote forms of *Leishmania* spp. [24–28] has made performing MST using a stage-specific axenic amastigote antigen (AMA) from different *Leishmania* species feasible and more appropriate. The amastigote form of this parasite is a biological stage that modulates human cellular and humoral immune responses against *Leishmania* infection. Considering the need for an accessible tool that can be extended to other regions of Brazil, where ACL is predominantly caused by *L.* (*Viannia*) spp. [11], using *L.* (*V.*) *lainsoni* AMA in MST may be promising because of not only its easy adaptation to laboratory culture media but also its exceptional capacity to induce a strong person’s cellular immune response [29, 30].

The present study aimed to evaluate the antigenic reactivity of *L.* (*V.*) *lainsoni* AMA for the laboratory diagnosis of ACL in the Brazilian Amazon using MST. Compared with that of the *L.* (*V*.) *braziliensis* promastigote (PRO), the results obtained using the new *L.* (*V.*) *lainsoni* AMA corroborate these aforementioned expectations, representing a new tool for strengthening the laboratory diagnosis of ACL in this region.

## Methods

### Study site

This prospective study was conducted from August 2020 to July 2022 at the RL/LL of the Parasitology Department, Evandro Chagas Institute, Secretary of Health and Environment Surveillance (Ministry of Health), Ananindeua, Pará State, Amazonian Brazil.

### Parasite strains

Two *L.* (*Viannia*) spp. strains were used to prepare the two antigens: *L.* (*V.*) *lainsoni* (MHOM/BR/1981/M6426/Benevides/Pará/Brazil) for AMA and *L.* (*V*.) *braziliensis* (MHOM/BR/1999/M17323/Paragominas/Pará/Brazil) for PRO.

Both antigens were obtained from the culture media: 199 culture plates containing a medium for *L.* (*V*.) *lainsoni* and RPMI for *L*. (*V*.) *braziliensis*. The two *L.* (*Viannia*) spp. were isolated from LCL [*L.* (*V*.) *lainsoni*] and ML [*L.* (*V.*) *braziliensis*] clinical forms, whose virulence was conserved and stored at the Evandro Chagas Institute biobank.

### Study population and clinical evaluation

Seventy individuals, comprising 60 ACL patients and 10 healthy individuals, were enrolled in this study. Patients, mainly from Pará, neighboring Brazilian states and French Guiana, who had visited the leishmaniasis outpatient clinic at the Evandro Chagas Institute, a regional referral center for the laboratory diagnosis and treatment of ACL, comprised the test group. Healthy individuals with no history of ACL residing in a non-endemic area made up the negative control group.

Laboratory investigations, such as direct parasitological and/or culture in Difco B_45_ medium [31] and serological assay (IFAT-IgG/cut-off: 80) also with *L.* (*V.*) *lainsoni* axenic amastigote antigen, were performed to confirm the diagnosis in all patients with clinical suspicion of ACL. These methods are used routinely at the RL/LL (histopathological and molecular approaches are subject to doubtful cases). Fifty-three patients with LCL and seven with ML were evaluated [6, 7, 17].

Clinical evaluation was performed based on the time course of the disease and type, number, and location of cutaneous and/or mucosal lesions [6, 7].

### Identification of *Leishmania* spp. in the patients

Parasites were preliminarily characterized based on their morphology and behavior in culture medium and in experimentally infected hamsters. Species determination was performed using a PCR-RFLP technique with two target sequences: RNA polymerase II gene, wherein the PCR products were cleaved with the TspRI and HgaI endonucleases, and *hsp*70 gene, wherein the PCR products were cleaved with HaeIII endonuclease. Restriction profiles were compared with those of the reference strains of the subgenera *L.* (*Viannia*) and *L.* (*Leishmania*), regarded as ACL agents in the Amazon region [32–34].

### Preparation of MST using *L.* (*V.*) *lainsoni *AMA and *L.* (*V.*) *braziliensis* PRO

*Leishmania* (*V.*) *lainsoni* promastigotes were cultured in Difco B45 medium for 7 days at 26 ºC until an optimal level of growth was achieved. It was subsequently seeded into 199 culture plates containing a medium (pH 7.2) supplemented with 0.36 g sodium bicarbonate (NaHCO^3^), 9.532 g Hepes, 0.1% biotin, 0.1% hemin, 0.5% adenine, 10% fetal bovine serum (FBS), 2% sterile human urine, and 1% penicillin/streptomycin and cultured for 3 days at 26 ºC until the logarithmic phase was reached.

The culture was transferred to 199 culture plates containing a medium (pH 7.2) supplemented with 25% FBS and incubated at 32 ºC (5% CO2) for 24 h.

The promastigote forms were centrifuged at 1562 g for 10 min at room temperature and resuspended in 199 culture plates containing a medium (pH 5.2) supplemented with l-glutamine, tryptone, glucose, 0.1% hemin, 1% adenine, 40% succinate buffer, 25% FBS, 2% sterile human urine, and 1% antibiotic and incubated for 5 days at 32 ºC (5% CO_2_) for differentiation into amastigote forms. The axenic amastigote forms were maintained at a ratio of one part culture/two parts 199 medium [35, 36].

The axenic amastigotes were washed thrice with sterile PBS after differentiation into 2–3 replicates and centrifuged at 1897 g for 10 min. The sediments containing amastigotes were resuspended, and the cell count was determined using a Neubauer chamber to prepare a final suspension at a concentration of 10^7^ cells/ml in thimerosal solution [ethyl(2-mercaptobenzoate-(2-)-O,S) mercurate(1-) sodium] at a concentration of 1/10,000, as previously used as a preservative and inactivating solution for promastigote antigens of *L*. (*L.*) *amazonensis* or *L.* (*V.*) *braziliensis* [9, 16, 22]. An aliquot of the cell concentrate was extracted before adding the preservative to evaluate (i) the protein concentration present in the AMA using the Bradford Method [37] and (ii) the integrity of axenic amastigote forms using optical microscopy and flow cytometry (BD FACSCANTO II™). The AMA was then aliquoted into 5-ml vials and refrigerated at 4 °C until further use.

Preparation of *L.* (*V.*) *braziliensis* PRO was performed under the same standard procedures and conservation processes as for *L.* (*L.*) *amazonensis* antigen [22] as well as the protein concentration by the Bradford method [37].

### MST using *L.* (*V.*) *lainsoni *AMA and *L.* (*V.*) *braziliensis* PRO

An intradermal injection of 0.1 mL AMA was administered on the anterior surface of the right forearm. An intradermal injection of 0.1 ml PRO was administered on the anterior surface of the left forearm. Thimerosal solution was also administered on the anterior surface of the right forearm as a control test to detect possible allergic reactions against the preservative used along with the antigens. Individuals reactive to thimerosal solution were excluded from the study owing to the lack of a definition of the specificity of the response to AMA or PRO.

Hypodermic syringes with 13 × 6.5 mm sterile disposable needles were used to administer the antigens and thimerosal solution intradermally. The tests were performed by two trained personnel with the same amount of experience to minimize individual errors. All individuals were observed for the first 30 min after the intradermal administration of the antigens and the thimerosal solution to detect immediate reactions. The injection sites were examined after 48 h to verify reactivity (induration, infiltration, or papuloerythematous reactions). The residual areas of reactivity were measured using a millimeter ruler, and the contours were marked using a ballpoint pen. Any cutaneous manifestation with a diameter of ≥ 5 mm was considered positive [6, 7].

### Data analyses

It was hypothesized that the MST reactivity against *L.* (*V.*) *lansoni* AMA would be greater than that against *L.* (*V*.) *braziliensis* PRO. A paired Student’s t-test was used to test this hypothesis as two dependent variables were present and to evaluate possible differences between the protein concentrations of the two antigens (AMA vs. PRO). The Shapiro-Wilk test was used to test the assumptions of the paired Student’s t-test [38].

Kappa agreement analysis was used [39] to determine the agreement between MST test results for *L.* (*V.*) *lainsoni* (AMA) and *L*. (*V*.) *braziliensis* (PRO). The kappa values were interpreted as follows: *K* = 0 indicated no agreement, whereas *K* ≠ 0 indicated agreement [40].

The correlation of the skin reaction diameter, in millimeters, between AMA and PRO antigens was determined using Spearman correlation test to assess their correspondence with the results of the kappa test. The assumptions of the correlation test were also tested using the Shapiro-Wilk test.

All statistical analyses were performed using RStudio software 1.3.3.3. (Rstudio Team, 2020). The “*dplyr*” [41] and *“corrplot”* packages were used [42] to analyze the results of the paired Student’s t-test and correlation analysis, whereas the “pacman” package was used to analyze the results of the kappa test [43]. The graphs illustrating the descriptive analyses were created using the “*ggplot2*” package [44]. The confidence interval was set at 95%, and *P* ≤ 0.05 indicates statistical significance.

## Results

### Morphological characteristics and viability of the parasites used to prepare *L*. (*V*.) *lainsoni *AMA and *L.* (*V.*) *braziliensis* PRO

The morphology and integrity of *L.* (*V.*) *braziliensis* and *L.* (*V.*) *lainsoni* were evaluated to ensure that antigens were obtained from intact cells and were analyzed via optical microscopy and flow cytometry. The promastigote forms of *L.* (*V.*) *braziliensis* are characterized by the presence of an elongated and fusiform body, nucleus, evident kinetoplast, and middle flagellum. The promastigote forms of *L.* (*V.*) *lainsoni* are characterized by the presence of a large, elongated, and fusiform cell body; evident nucleus; kinetoplast; and typical long flagellum, which was larger than the body. The axenic amastigote forms of *L.* (*V.*) *lainsoni* presented as small cells without flagella, ovoid bodies, nuclei, or kinetoplasts.

Flow cytometry using propidium iodide (PI) labeling revealed that 72.3% of *L.* (*V*.) *lainsoni* axenic amastigotes were viable.

### Protein concentration of *L.* (*V.*) *lainsoni *AMA and *L.* (*V.*) *braziliensis* PRO

Analysis of the protein concentration of *L.* (*V.*) *lainsoni* AMA and *L.* (*V.*) *braziliensis* PRO using the Bradford method revealed that the concentrations of AMA and PRO were 173.3 μg/ml (equivalent to 17.3 ng/ml/antigen dose in 0.1 ml) and 241.2 μg/ml (equivalent to 24.1 ng/ml/dose in 0.1 mL), respectively, indicating that the protein concentrations of both were similar (t-test,* t*_(3)_ = − 2.6185, *P* = 0.0588).

### Laboratory diagnosis of ACL for the examined patients

Among the 60 patients examined at the RL/LL presenting clinical suspicious of ACL, a direct parasitological and/or culture and serological (IFAT-IgG) diagnosis was confirmed in 46 (76.6%), comprising 43 (81.1%) with LCL and 3 (43%) with ML, while serological diagnosis was confirmed alone in 10 (18.9%) patients with LCL and 4 (57%) with ML.

### Personal and clinical characteristics of the study population

Sixty patients (100% male) with a laboratory-confirmed diagnosis of ACL were included in this study. Fifty-three (88.3%) and seven (11.7%) patients presented clinical features of LCL and ML (G1 group), respectively. Ten healthy individuals [4 males (40%) and six females (60%)] with no history of ACL formed the negative control group (G2). The mean age of patients with LCL was 41 years, while in those with ML it was 43; in the control group, it was 38.5.

### Clinical evaluation of the examined patients

Average disease duration in the 53 patients with LCL was 4.4 months. Average disease duration in the seven patients with ML was 83 months (approximately 7 years). The predominant type of skin lesion was ulcerated lesions (≥ 98%) in patients with LCL; 2–3 lesions per patient were observed on average, mainly on the upper and lower limbs. In contrast, the mucosal lesion had a granulomatous appearance and affected the nasobuccopharyngeal region in four (57%) of the seven patients with ML.

### Identification of *Leishmania* spp. from the examined patients

Among the 43 LCL patients with a parasitological diagnosis, *Leishmania* spp. was isolated from 32 samples (one sample from each patient) whose molecular characterization revealed 31 belonging to *L.* (*Viannia*) subgenus: *L.* (*V.*) *braziliensis* (*n* = 9), *L.* (*V.*) *shawi* (*n* = 6), *L.* (*V*.) *lainsoni* (*n* = 5), *L.* (*V.*) *lindenbergi* (*n* = 3), *L.* (*V*.) *guyanensis* (*n* = 2), and *L.* (*V.*) *naiffi* (*n* = 1). Five samples could not be defined completely. *Leishmania* (*L.*) *amazonensis* was detected in the last sample. Among the three ML cases with a parasitological diagnosis, *L.* (*V.*) *braziliensis* was isolated from two. Thus, among the 60 patients examined, *Leishmania* spp. were isolated from 34 (56.6%): *L.* (*Viannia*) subgenus (*n* = 33) and *L.* (*Leishmania*) subgenus (*n* = 1).

### *Leishmania* (V.) *lainsoni* AMA was more reactive than *L*. (*V*.) *braziliensis* PRO

Among the 60 patients with ACL examined in the present study, a positive MST reaction to AMA and PRO was observed in 60 (100%) and 59 (98.3%) patients, indicating similar sensitivity to both antigens. However, comparison of the amplitude of MST antigenic reactivity (diameter of skin reactions in mm) against these antigens revealed that the mean (mean ± standard deviation) for AMA (19.4 mm ± 13.3) was higher than that for PRO (12.1 mm ± 8.1), which was also evident when these results were compared according to the clinical forms of the disease, i.e. LCL (18.8 mm ± 13.3 × 11.8 mm ± 8.2) and ML (24.3 mm ± 13.7 × 14.6 mm ± 8.4), respectively.

**Fig. 1:**
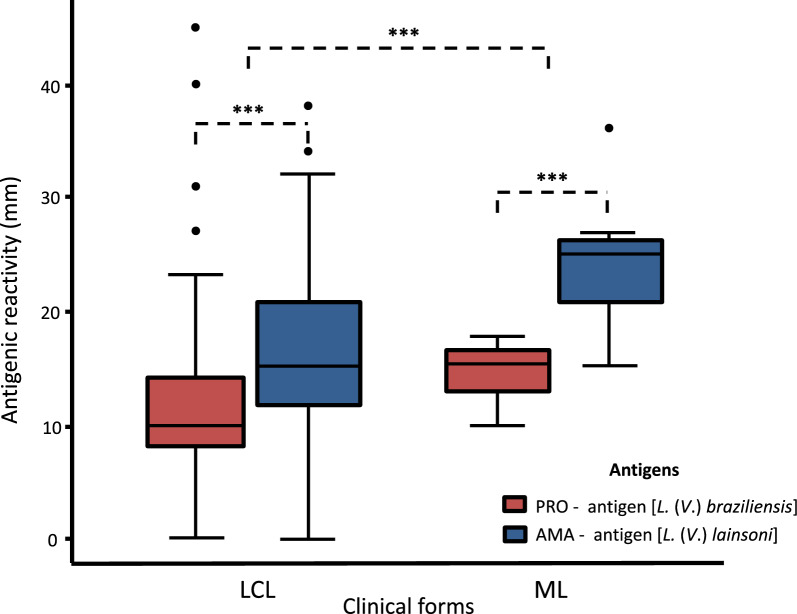
Montenegro skin test reactions (in millimeters) from ACL patients submitted to laboratory diagnosis. AMA antigen: *Leishmania* (*V*.) *lainsoni* axenic amastigote antigen; PRO antigen: *L.* (*V.*) *braziliensis* promastigote antigen. LCL: localized cutaneous leishmaniasis. ML: mucosal leishmaniasis. ***P < 0.001

Paired Student’s *t*-test revealed that the mean MST reactivity against AMA differed from that against PRO (t-test,* t*_(63)_ = − 8.5224, *P* < 0.001), indicating that the average reactivity against AMA was greater than that against PRO. Evaluation of MST reactivity according to the clinical forms of ACL revealed that patients with LCL and ML exhibited a significantly stronger reaction against AMA (t-test, *t*_(56)_ = − 7.5619; *P* < 0.001 and t-test, *t*_(6)_ = − 4.7532; *P* = 0.0031), respectively (Table 1; Fig. 1). Individuals in the control group did not exhibit reactivity to AMA or PRO.

**Table 1:** Montenegro skin test reactions [mean and standard deviation (SD)] of *L.* (*V.*) *lainsoni* AMA and *L.* (*V.*) *braziliensis* PRO antigens according to ACL clinical forms

	^a^LCL	^b^MCL	Total
PRO	11.8 (8.2)	14.6 (8.4)	12.1 (8.1)
AMA	18.8 (13.3)	24.3 (13.7)	19.4 (13.3)
*P*-value	< 0.001	< 0.001	< 0.001
t-test	*t*_(56)_ = − 7.5619	*t*_(6)_ = − 4.7532	*t*_(63)_ = − 8.5224

^a^LCL: localized cutaneous leishmaniasis

^b^MCL: mucosal leishmaniasis

Although the 60 ACL patients and ten healthy individuals (control group) had each received three intradermal injections (0.1 ml), two with the AMA and PRO antigens and one with the thimerosal solution, during the first 30 min, none manifested any local sign and/or systemic symptom of significant magnitude, that is, discomfort (irritation or itching) or local pain, general malaise, drowsiness, dizziness, or syncope. Upon return (after 48 h), only three (48%) of the seven ML patients, those with reactions ≥ 15 mm in diameter, reported moderate hyperthermia (up to 37.6 °C) and/or headache lasting up to 24 h at ~ 12 h after the injections; at the time of assessment of intradermal reactions, there were no more complaints of these side reactions.

### Diagnostic agreement and correlation between AMA and PRO

Kappa agreement analysis revealed a strong reliability between AMA and PRO antigens [*k* = 0.94 (95% CI 0.88–1.00)] and a diagnostic agreement of 98.6%. Thus, the antigens were concordant in 69 of the 70 individuals tested (60 patients with ACL and 10 negative controls). One individual was positive only for AMA and negative for PRO (Table 2).

**Table 2:** Diagnostic agreement between AMA and PRO antigens used by MST in the laboratory diagnosis of ACL (60 patients) and 10 healthy individuals (control group)

	AMA	
	Positive	Negative
PRO		
Positive	59 (0.843)	0 (0)
Negative	1 (0.014)	10 (0.143)
Agreement	98.6%	
*Kappa*	0.94 (IC 95%: 0.88–1.00)	
Conclusion	Perfect	
*P*-value (unilateral)	< 0.001	

AMA: *Leishmania* (*V*.) *lainsoni* axenic amastigote antigen; PRO: *L.* (*V.*) *braziliensis* promastigote antigen

**Fig. 2:**
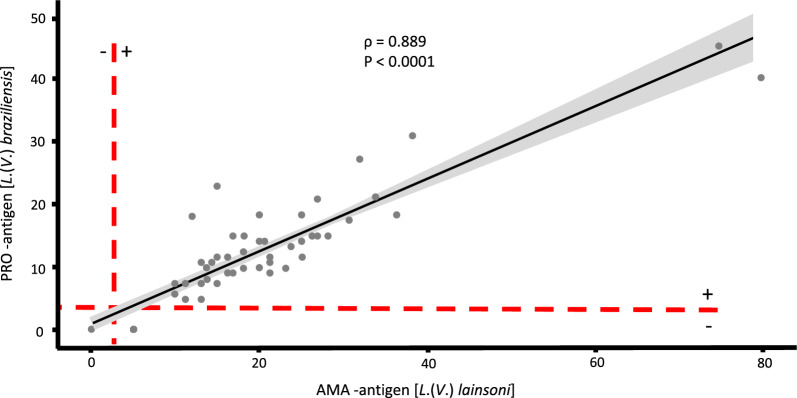
Spearman correlation of Montenegro skin test reactivities (diameter, in millimeters) between AMA and PRO antigens in the test (60 ACL-confirmed patients) and control group (10 healthy individuals)

The Spearman test revealed a positive correlation between AMA and PRO (rho = 0.889, *P* < 0.0001) (Fig. 2).

## Discussion

In this study we examined the antigenic reactivity of the AMA of *L. (V.) lainsoni*, an important etiologic agent of ACL in Latin America [36, 45–49], as an alternative antigen for optimizing MST in the laboratory diagnosis of the disease.

The MST results of the 70 participants tested (60 patients with ACL, comprising 53 with LCL and 7 with ML, and 10 healthy individuals) revealed that the antigenic reactivity of *L.* (*V.*) *lainsoni* AMA was significantly higher than that of *L.* (*V.*) *braziliensis* PRO. This finding was also true in the analysis of the antigenic reactivity according to the clinical forms of ACL, i.e. LCL and ML, respectively. These findings are of great technical and scientific interest, especially since the protein concentrations of both antigens were similar. Thus, the difference in reactivity may be attributed to the greater specificity of *L.* (*V.*) *lainsoni* AMA compared with that of *L.* (*V*.) *braziliensis* PRO, confirming the good stability, such as integrity and cellular viability, of AMA.

Although the antigens used in this study are not species specific, but subgenus specific, i.e. *L.* (*Viannia*) *lainsoni* × *L.* (*Viannia*) *braziliensis*, the above data appear to confirm that the AMAs of the parasite are more specific than those of PROs as the amastigote stage of the parasite interacts directly with the person’s cellular and humoral immune responses following the establishment of the infection [3, 11]. Furthermore, the complete lack of MST response in the 10 healthy individuals with no history of ACL (control group) appears to confirm the specificity of both antigens.

Analysis of the sensitivity of AMA and PRO revealed rates of 100% and 98.3%, respectively, indicating similar rates. This finding highlights the great capacity of these antigens to recognize the specific DTH response observed in patients with ACL. However, the rates were not identical (100% for both), as MST reactivity for *L.* (*V.*) *braziliensis* PRO was not observed in the patient with LCL caused by *L. (L.) amazonensis*. This finding has already been recorded with high frequency (≥ 80%) for the current *L.* (*V.*) *braziliensis* PRO and a species-specific antigen of *L.* (*L.*) *amazonensis* PRO [7, 9, 10, 16], reinforcing the intriguing ability of *L.* (*L.*) *amazonensis* to escape the human cellular immune response [50–52]. However, this finding also highlights the specificity of *L*. (*V.*) *lainsoni* AMA in recognizing DTH even in patients with LCL caused by *L.* (*L.*) *amazonensis*, although it was observed in only one case examined in the present study (which PRO failed to recognize).

The sensitivity rates of AMA and PRO recorded in the present study are among the highest (≥ 98%) when compared with those recorded by previous studies conducted in Brazil and Argentina (South America), such as Rio de Janeiro, ≥ 90% [53]; São Paulo, 82–89% [12]; Paraná, 84.4% [54]; Ceará, 46.3% [55]; and a retrospective analysis conducted at a reference center in Argentina (also ≥ 90%) [56]. The Department of Surveillance of Communicable Diseases of the Secretariat of Health and Environmental Surveillance of the Ministry of Health of Brazil [57] revealed the reference rate of MST sensitivity is approximately 90%. Thus, the rates observed in the present study for AMA (100%) and PRO (98.3%) indicate more robust results than those reported by different sources.

MST reactivity against AMA [*L.* (*V*.) *lainsoni*] and PRO [*L.* (*V.*) *braziliensis*] in patients with LCL and ML in this study showed that the reactivity against AMA was higher than that against PRO in both clinical forms, LCL and ML; however, stronger MST expression was observed in ML than LCL. This finding can be attributed to the distinct immunopathological mechanisms involved in the development of these clinical forms of ACL. The T-cell immune response, mainly of the CD4^+^/Th1-type (which is strongly associated with DTH), is more activated in ML than LCL [9, 10, 58, 59]. Furthermore, the duration of disease was longer in ML (approximately 7 years) than LCL (4.4 months), indicating a longer lasting immune stimulus in patients with ML than in those with LCL. This promotes a much stronger MST response in patients with ML. However, this does not seem to remember that, in addition to the action of the parasite’s species-specific antigens [3, 11], human genetic factors [60–62] and even co-infection of *L.* (*V.*) *braziliensis* with *Leishmania* RNA virus 1 [48, 63] may be involved in modulating these immunopathological mechanisms in ML.

Regarding the higher expression of antigenic reactivity against AMA, notably, diagnostic agreement analysis showed a high rate (98.6%) and Spearman analysis evidenced a strong positive correlation, indicating that these antigens have similar potential for use in the laboratory diagnosis of ACL, i.e. either antigen can be used in the absence of the other. However, this raises a question regarding the advantage of replacing the *L.* (*V*.) *braziliensis* PRO with *L.* (*V*.) *lainsoni* AMA. The advantage is not necessarily in replacing PRO with AMA but in knowing that today a new antigen (AMA) is available that expresses greater reactivity in the laboratory diagnosis of ACL caused by all *Leishmania* spp. of *L.* (*Viannia*) subgenus occurring in Brazil [*L.* (*V.*) *braziliensis*, *L.* (*V.*) *guyanensis*, *L.* (*V.*) *shawi*, *L.* (*V.*) *lainsoni*, *L.* (*V.*) *lindenbergi*, and *L.* (*V.*) *naiffi*] and possibly caused by *Leishmania* spp. of *L.* (*Leishmania*) subgenus, such as *L.* (*L.*) *amazonensis* [8, 11], as shown here (and perhaps in Latin America).

This finding is particularly useful as Brazil and most Latin American countries do not have a similar PRO owing to the lack of industrial infrastructure compatible with that of Good Manufacturing Practices (GMP) for the production of MST antigen and the low commercial profitability of industrial production of antigen [64, 65]. Additionally, this technique has shown potential for detecting ACL due to *Leishmania* spp. of *L.* (*Leishmania*) subgenus, specifically, *L.* (*L*.) *amazonensis*, which has demonstrated great capacity to evade cellular immunity [50–52]. These findings indicate that investing in large-scale AMA antigen productivity, in addition to optimizing MST for laboratory diagnosis of ACL, will aid in addressing the demand from Brazil and neighboring countries.

Even though these findings revealed that AMA antigen represents a promising alternative for optimizing MST in the laboratory diagnosis of ACL, future studies must seek to elucidate the composition and immunogenicity of AMA proteins to develop a synthetic antigen applicable to all epidemiological scenarios of the disease in Latin America. It has been clearly demonstrated that the use of AMA from *L*. (*V.*) *lainsoni*, the most ancestral *Leishmania* species of *L*. (*Viannia*) subgenus [66–68] that carries a protein genetic load common to all *Leishmania* spp. of this subgenus, would represent a desirable major advance in the laboratory diagnosis of ACL.

The crucial role of MST in defining the clinical-immunopathological spectrum of ACL in the Brazilian Amazon should also be highlighted [2, 3, 9–11, 16, 17], where a moderately positive MST in LCL is caused by *Leishmania* spp. of *L.* (*Viannia*) subgenus, especially *L.* (*V.*) *braziliensis*. This reactivity increases as the infection evolves toward the hyperergic pole (CD4^+^/Th1-type) of that spectrum represented by ML. In contrast, the reactivity to MST in LCL caused by *L.* (*L*.) *amazonensis* is weak or negative and completely regresses as the infection progresses to the hypoergic pole (CD4^+^/Th2 type) of the ACL spectrum represented by severe and incurable ADCL. Thus, MST can also be used as a prognostic marker for the treatment of the different clinical forms within the clinical-immunopathological spectrum of ACL, which can be performed with a higher degree of accuracy using AMA antigen as it showed greater fidelity to the cellular immune response (DTH) of patients with LCL and ML examined here.

Lastly, as mentioned before, it has been almost 50 years since the RL/LL introduced the MST as a complementary tool for the laboratory diagnosis of ACL. During this period, two antigenic compositions were used to perform MST, with promastigote of *L.* (*L*.) *amazonensis* [22] and *L.* (*V*.) *braziliensis* [3, 9, 10], without records of any incident (serious side effect) resulting from use of these antigenic compositions. The results presented now demonstrated that, although a different methodology from the previous one was introduced for the production of the new antigen [*L.* (*V*.) *lainsoni* AMA] used to perform MST, no serious side effects, local or systemic, were recorded in 70 individuals tested (60 ACL patients and 10 healthy individuals), confirming the GMP used for the preparation of these antigens.

## Conclusions

The studied patients exhibited a higher reactivity to *L.* (*V.*) *lainsoni* AMA than to *L.* (*V.*) *braziliensis* PRO in the MST. This indicates that the *L.* (*V*.) *lainsoni* AMA is a promising alternative for optimizing MST in the laboratory diagnosis of ACL.

## Data Availability

All data supporting the main findings of this study are found in the manuscript.

## Abbreviations

ACL: American cutaneous leishmaniasis
MST: Montenegro skin test
DTH: Delayed-type hypersensitivity response
LCL: Localized cutaneous leishmaniasis
BDCL: Borderline disseminated cutaneous leishmaniasis
ML: Mucosal leishmaniasis
ADCL: Anergic diffuse cutaneous leishmaniasis
AMA: Axenic amastigote antigen
PRO: Promastigote antigen
PCR: Polymerase chain reaction
RL/LL: Ralph Lainson Leishmaniases Laboratory
RPMI: Roswell Park Memorial Institute
IFAT: Indirect fluorescent antibody test
IgG: Immunoglobulin G
RFLP: Restriction fragment length polymorphism
RNA: Ribonucleic acid
*hsp*: Heat shock protein
CD4/Th1: Lymphocyte CD4/T-helper type 1
CD4/Th2: Lymphocyte CD4/T-helper type 2
